# Dietary Omega-3 intake may slow prostate cancer progression and reduce mortality risk: evidence from prostate, lung, colorectal, and ovarian cancer screening trial

**DOI:** 10.3389/fnut.2025.1623295

**Published:** 2025-07-21

**Authors:** Xinru Shu, Jiayi Lin, Ling Yao, Shun Liu, Qingquan Chen, Honggeng Wang, Liangming Wang

**Affiliations:** ^1^The Second Affiliated Hospital of Fujian Medical University, Quanzhou, Fujian Province, China; ^2^Fujian Medical University, Fuzhou, China

**Keywords:** prostate cancer, Omega-3, docosahexaenoic acid, docosapentaenoic acid, eicosapentaenoic acid, diet, restricted cubic spline

## Abstract

**Background:**

Prostate cancer is the second most prevalent malignant cancer globally and the fifth leading cause of cancer-related mortality. Omega-3 PUFAs [including eicosapentaenoic acid (EPA), docosapentaenoic acid (DPA), and docosahexaenoic acid (DHA)] may mitigate prostate cancer risk through various molecular mechanisms, such as inhibiting pro-inflammatory eicosanoid production via COX-2 pathway, regulating apoptosis and autophagy, and potentially influencing other signaling pathways like NF-κB. However, existing studies have reported inconsistent findings regarding the effects of Omega-3 fatty acids on prostate cancer, and there is limited large-scale longitudinal data exploring the dose-response relationship.

**Methods:**

This study utilized data from the Prostate, Lung, Colorectal, and Ovarian (PLCO) Screening Trial to investigate the associations between Omega-3 (EPA, DPA, DHA) intake from dietary sources and the risk of prostate cancer and mortality. Omega-3 fatty acid intake was assessed via the Dietary History Questionnaire (DHQ). Multivariate Cox proportional hazards regression models, adjusted for key confounders including age, race, BMI, family history, PSA levels, comorbidities, and lifestyle factors, were employed alongside restricted cubic spline (RCS) analysis to account for potential confounding factors. A total of 30,552 male participants aged 55–74 years were included, with a median follow-up time of ≥7 years.

**Results:**

The results indicated a linear relationship between Omega-3 fatty acid intake and the overall risk of prostate cancer (HR for highest vs. lowest quintile: 0.90, 95% CI: 0.81–1.00, *P* = 0.053). For prostate cancer mortality, a non-linear relationship was observed (*P*_non − linearity_ = 0.009). The risk of death decreased as intake increased below 0.4 g/d, with a hazard ratio of 0.67 (95% CI: 0.49–0.91, *P* = 0.011) for the second quintile compared to the lowest quintile. However, intake exceeding this threshold was associated with an increased risk (HR for highest quintile: 0.70, 95% CI: 0.52–0.95, *P* = 0.021). The RCS analysis revealed a potential U-shaped association for mortality risk, with the lowest risk corresponding to an intake range of ~0.15–0.40 g/d.

**Conclusion:**

Increased dietary intake of Omega-3 fatty acids has been associated with a reduced risk of prostate cancer. However, the association with mortality risk showed a threshold effect, with intakes below 0.4 g/day reducing mortality and higher intakes potentially increasing risk.

## Introduction

Prostate cancer is a malignant cancer originating from the male reproductive system. It is currently the second most common cancer in men worldwide, second only to lung cancer, and the fifth leading cause of cancer death. Age is one of its recognized risk factors, especially affecting men over 60 years old (1, 2). In addition to age, the main known risk factors for prostate cancer include race, obesity, and family history (3). There are also some modifiable risk factors, such as dietary intake, physical labor, and sleep (1). It is worth noting that dietary intake can be analyzed digitally through daily eating habits, so dietary intervention may become an adjuvant treatment for prostate cancer.

The European Prospective Investigation into Cancer and Nutrition (EPIC) study demonstrated an association between the risk of high-grade prostate cancer and the intake of total fat, monounsaturated fat, and polyunsaturated fat (4). In terms of dietary intake, studies have shown that Omega-3 polyunsaturated fatty acids (PUFA) have a protective effect on prostate cancer survival rate (5). Omega-3 fatty acids can be obtained through the consumption of the liver from lean white fish such as cod and halibut; the flesh of oily fish, including mackerel, herring, and salmon; and widely used fish oil supplements (6). Omega-3 is a type of substance that is currently recognized to have anti-inflammatory effects. It exerts anti-inflammatory effects through multiple pathways, such as reducing the synthesis of pro-inflammatory mediators, regulating cell signal transduction processes, and activating transcription factors such as PPAR-γ, inhibiting the activation of the NF-κB signaling pathway, thereby reducing the expression of inflammation-related genes (7). A study by Richard J. Deckelbaum revealed that Omega-3 fatty acids can reduce the occurrence and development of cardiovascular disease, diabetes, cancer and other diseases by changing cell membrane fluidity, regulating nuclear receptors and inhibiting inflammatory mediators (8). The components of Omega-3 fatty acids include eicosapentaenoic acid (EPA), docosapentaenoic acid (DHA) (these two exist in marine animal and plant oils) and docosapentaenoic acid (DPA). EPA and DHA are currently considered the most biologically active forms of Omega-3 fatty acids; DPA, as an intermediate of EPA and DHA, may contribute to the health of the body (9). Continuous intake of oily fish (rich in Omega-3) is associated with a reduced risk of prostate cancer. The mechanism by which Omega-3 fatty acids prevent carcinogenesis involves the inhibition of eicosanoids synthesized from arachidonic acid to reduce inflammatory responses, thereby potentially delaying the progression of prostate cancer (10). In addition, Omega-3 fatty acids also exert anticancer effects through mechanisms such as the regulation of apoptosis and autophagy (7, 11).

In a study on the correlation between Omega-3 fatty acids and atrial fibrillation, Frank Qian et al. investigated the relationships between the levels of EPA, DPA, and DHA in blood or adipose tissue and atrial fibrillation (12). Therefore, this research studies the calculation of Omega-3 content as the basis for determining total Omega-3 intake; that is, the daily intake of EPA, DPA and DHA is used to express Omega-3 intake.

Given the anti-inflammatory properties of Omega-3 fatty acids and their potential to inhibit cell growth factors, researchers have hypothesized that an increased intake of Omega-3 fatty acids from either food or supplements might lead to a reduced risk of cancer (13). This hypothesis has spurred extensive research into the correlation between Omega-3 fatty acids and cancer. However, the National Institutes of Health (NIH) in the United States currently harbors considerable controversy regarding the relationship between Omega-3 fatty acid intake and cancer, as presented in the professional version of Omega-3 fatty acid. In a nested case–control analysis of men aged 55–84 years in a prostate cancer prevention trial, serum DHA phospholipid levels were positively correlated with the risk of high-grade prostate cancer but not with the risk of low-grade prostate cancer (14). Similarly, the results from a case–cohort study in the Selenium and Vitamin E Cancer Prevention (SELECT) trial also revealed that men with higher Omega-3 levels had a greater risk of prostate cancer (15). Though higher serum Omega-3 levels were associated with increased high-grade prostate cancer risk, causality was not established, and the source of Omega-3 remains unclear. However, some studies have shown that dietary intake of Omega-3 fatty acids has no effect on the risk of prostate cancer, while eating fish reduces prostate cancer mortality but has no effect on prostate cancer incidence (16). Another meta-analysis on Omega-3 fatty acids and prostate cancer also revealed that there was no significant association between dietary intake or blood Omega-3 fatty acids and the overall risk of prostate cancer (17). In addition to the above two results, other observational studies using dietary intake data from other scholars have shown that higher intake of fish and/or Omega-3 fatty acids may reduce the risk of prostate cancer.

Therefore, the relationship between Omega-3 intake and prostate cancer remains a controversial topic. On the one hand, the potential mechanism of Omega-3′s anti-inflammatory and antioxidant properties is still uncertain. On the other hand, there is limited data related to the two, which leads to insufficient recommendations and understanding about Omega-3 intake. There are few nutritional recommendations that can provide clear evidence for dietary intervention treatment of prostate cancer. In addition, there are relatively few research teams that study the effects of Omega-3 on prostate cancer in the context of large-scale population data.

This study was based on the Prostate, Lung, Colorectal, and Ovarian Cancer (PLCO) Screening Trial Database to evaluate whether dietary intake of Omega-3 (EPA, DHA, and DPA) is associated with the incidence and mortality of prostate cancer. The results of this study can reduce the incidence of prostate cancer in healthy people, help optimize dietary behavior and promote health, and provide dietary-assisted treatment recommendations for prostate cancer patients to improve prognosis and quality of life.

## Method

### Study design

The dataset utilized in this investigation was sourced from the PLCO study (https://cdas.cancer.gov/plco/). The PLCO Cancer Screening Trial is a large, population-based, randomized controlled trial designed to evaluate the effectiveness of cancer screening in men and women aged 55–74 years and whether early detection can reduce cancer-related deaths (18). A total of 154,887 participants were enrolled in the trial from November 1993 to July 2001, including 76,678 men and 78,209 women. Throughout the trial, the participants were required to complete a series of questionnaires. The baseline questionnaire (BQ) provided background information and was completed by 97% of the participants before the start of the trial. Two dietary questionnaires (DQX for the screening group and DHQ for both groups) were potentially completed in the first few years of follow-up to record self-reported dietary habits. A supplementary questionnaire (SQX) was completed in the later stages of the trial to obtain information similar to the BQ. The DHQ questionnaire mainly records information such as the daily intake frequency, daily intake, cancer incidence time, and death exit time of participants for various foods during the follow-up period.

### Study population

**Figure 1:**
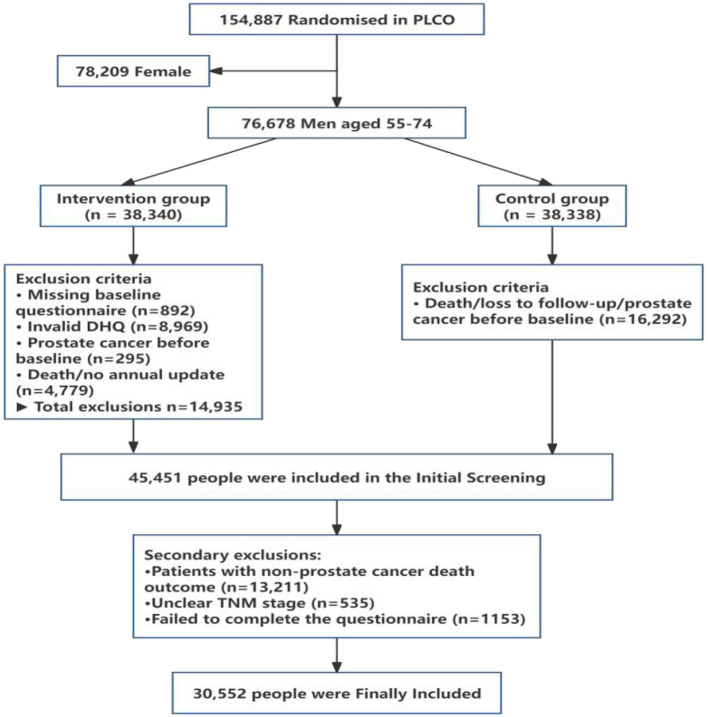
PLCO participant flow chart.

A total of 76,678 men aged 55–74 years, out of the 154,887 participants enrolled, were randomly allocated to either the intervention group (*n* = 38,340) or the control group (*n* = 38,338) in the PLCO trial. In the present study, only men were considered because the outcome of the study was prostate cancer. The participants in both groups completed a dietary history questionnaire (DHQ). Among all the men assigned to the intervention group, 14,935 individuals were excluded on the basis of the following screening criteria: absence of a baseline questionnaire (*n* = 892), invalid or incomplete DHQ (*n* = 8,969), preexisting prostate cancer diagnosis prior to the study commencement (*n* = 295), and death or failure to provide annual study updates (*n* = 4,779). Of the 38,338 men in the control group, 16,292 men were excluded from the PLCO control group because of death, loss to follow-up, or prevalent cases of prostate cancer before completing the DHQ. Among the 46,309 participants identified after screening, a group of participants were excluded because they were non-prostate cancer patients with death outcomes (*n* = 13,211), unclear TNM stages (*n* = 535) or incomplete questionnaires (*n* = 1,153). The final number of participants included in the study was 30,552, including non-advanced prostate cancer patients (*n* = 2,969) and advanced prostate cancer patients (*n* = 388). The detailed process of inclusion and exclusion can be seen in Figure 1.

Written informed consent was obtained from all participants to participate in the PLCO study, which was approved by the Institutional Review Board of the National Cancer Institute (NCI) of the United States. The project ID is PLCO-1221.

### Assessment of prostate cancer

In the PLCO Screening Trial, men in the intervention group underwent annual prostate-specific antigen (PSA) testing and digital rectal examination (DRE) for the first 6 years, with all participants followed for a minimum of 7 years for prostate cancer outcomes. Diagnosis was ascertained primarily through medical record review for men flagged by: (i) self-reported prostate cancer during annual follow-up, (ii) abnormal PSA levels (>4 ng/mL, per clinical guidelines at the time), (iii) abnormal DRE findings, or (iv) prostate cancer indicated on a death certificate. Advanced prostate cancer was defined as a clinical stage III or IV cancer (based on TNM classification) or a cancer with a Gleason score ≥8 (19).

### Dietary assessment

The Dietary History Questionnaire (DHQ), a food frequency questionnaire (FFQ), was developed by the staff of the Risk Factor Monitoring and Methods Branch (RFMMB). The FFQ contains 124 food items, including portion size and dietary supplement questions. Moreover, the food list and nutrient database used by the DHQ are based on national dietary data (USDA Continuing Survey of Food Intake by Individuals (CSFII) 1994–1996, available from the USDA Food Survey Research Group: https://www.icpsr.umich.edu/web/ICPSR/studies/21960). The DHQ questionnaire was utilized to gather data on the average daily intake of DHA, DPA, and EPA, which were subsequently summed to represent the average daily Omega-3 intake.

### Statistical analysis

The baseline characteristics of the subjects were compared across different quintiles of Omega-3 (EPA, DPA, DHA) intake, employing chi-square tests for categorical variables and analysis of variance for continuous variables. Subjects with any or advanced prostate cancer were compared with subjects without such malignancies during follow-up by Omega-3 (EPA, DPA, DHA) intake, which was calculated via the DHQ. The Wilcoxon rank sum test was used for these comparisons. Hazard ratios (HRs) and their 95% confidence intervals (CIs) for the association between Omega-3 (EPA, DPA, DHA) intake and prostate cancer risk were estimated via Cox proportional hazards regression models with multivariate adjustment for important covariates, including known or suspected prostate cancer risk factors such as age, race, education, marital status, family history of prostate cancer, randomized trial subgroups, BMI, height, history of diabetes, history of vasectomy, smoking in the past 10 years, and energy-adjusted lycopene, calcium, and vitamin E intake. Omega-3 intake was divided into quintiles based on the entire sample, and HRs for quintiles 2–5 were calculated using the first (lowest) quintile as a reference. Linear trends across quintiles of Omega-3 fatty acid intake were tested by modeling the median of each quintile as a continuous variable via Cox regression.

To examine the potential non-linear dose–response relationship between Omega-3 fatty acid content and both prostate cancer incidence and the risk of prostate cancer death, Omega-3 fatty acid intake was modeled as either a categorical or continuous variable, utilizing restricted cubic splines (RCSs) with three knots (i.e., the 10th, 50th, and 90th percentiles), and chi-square tests for categorical variables and analysis of variance for continuous variables were applied. In sensitivity analyses, cancer cases occurring within the first 2 years of follow-up were excluded to minimize reverse causality. All the statistical analyses were performed via R (version 4.2.3), and *P* < 0.05 was considered statistically significant.

## Results

The baseline characteristics of the subjects are presented in Table 1. On the basis of the established inclusion and exclusion criteria, a total of 30,552 male subjects were ultimately included in the study. Omega-3 fatty acid intake was categorized into five quintiles (Q1–Q5) via the five-point method. Significant differences were observed across the quintiles of Omega-3 (EPA, DPA, DHA) intake in terms of age, education, marital status, height, BMI, race, lycopene intake, calcium intake, and vitamin E intake (*P* < 0.001). Additionally, statistically significant differences were noted in the prevalence of diabetes and history of vasectomy (*P* < 0.05).

**Table 1:** Baseline characteristics of study subjects by dietary intake of total Omega-3 in the PLCO screening trial.

**Variable**	**Dietary Intake of Total Omega-3 (EPA, DPA, DHA) (g/d)**						***P***
	**Overall (*****n** =* **30,552)**	**Q1 (** ≤ **0.04) (*****n** =* **7,650)**	**Q2 (>0.04 to** ≤ **0.06) (*****n** =* **4,600)**	**Q3 (>0.06 to** ≤ **0.09) (*****n** =* **6,158)**	**Q4 (>0.09 to** ≤ **0.15) (*****n** =* **6,236)**	**Q5 (>0.15 to** ≤ **1.94) (*****n** =* **5,878)**	
Age, years	60.9 ± 4.6	61.6 ± 4.8	61.1 ± 4.7	60.9 ± 4.6	60.7 ± 4.5	60.3 ± 4.4	**< 0.001**
**Education, %**							
Less than high school	7,207 (23.6)	2,172 (28.4)	1,177 (25.6)	1,397 (22.7)	1,323 (21.2)	1,138 (19.4)	**< 0.001**
High school graduate or equivalent	3,781 (12.4)	1,054 (13.8)	622 (13.5)	720 (11.7)	751 (12.0)	634 (10.8)	
Post-high school education	5,909 (19.4)	1,557 (20.4)	919 (20.0)	1,225 (19.9)	1,167 (18.7)	1,041 (17.7)	
College graduate or higher	13,625 (44.6)	2,867 (37.5)	1,882 (40.9)	2,816 (45.7)	2,995 (48.0)	3,065 (52.1)	
**Marital, %**							
Married	26,630 (87.2)	6,616 (86.5)	4,075 (88.6)	5,416 (88.0)	5,499 (88.2)	5,024 (85.5)	**< 0.001**
Married but living as single	3,043 (10.0)	807 (10.5)	417 (9.1)	584 (9.5)	572 (9.2)	663 (11.3)	
Single	849 (2.8)	227 (3.0)	108 (2.3)	158 (2.6)	165 (2.6)	191 (3.2)	
Height, inch	70.1 ± 2.7	69.9 ± 2.7	70.0 ± 2.6	70.1 ± 2.7	70.1 ± 2.7	70.2 ± 2.8	**< 0.001**
BMI, kg/m^2^	27.4 ± 3.9	27.2 ± 3.7	27.4 ± 3.8	27.4 ± 3.9	27.6 ± 4.0	27.7 ± 4.1	**< 0.001**
**Race, %**							
White, Non-Hispanic	27,725 (90.8)	6,905 (90.3)	4,263 (92.7)	5,673 (92.1)	5,706 (91.5)	5,178 (88.1)	**< 0.001**
Black, Non-Hispanic	738 (2.4)	203 (2.7)	90 (2.0)	122 (2.0)	139 (2.2)	184 (3.1)	
Hispanic	595 (1.9)	209 (2.7)	102 (2.2)	97 (1.6)	103 (1.7)	84 (1.4)	
Asian	1,246 (4.1)	299 (3.9)	120 (2.6)	225 (3.7)	246 (3.9)	356 (6.1)	
Others	218 (0.7)	34 (0.4)	25 (0.5)	41 (0.7)	42 (0.7)	76 (1.3)	
**Cigarette smoking, %**							
Never smoked cigarettes	12,619 (41.3)	3,198 (41.8)	1,897 (41.2)	2,577 (41.8)	2,550 (40.9)	2,397 (40.8)	0.262
Current cigarette smoker	2,386 (7.8)	622 (8.1)	373 (8.1)	493 (8.0)	474 (7.6)	424 (7.2)	
Former cigarette smoker	15,517 (50.8)	3,830 (50.1)	2,330 (50.7)	3,088 (50.1)	3,212 (51.5)	3,057 (52.0)	
**Family history of prostate cancer, %**							
No	28,192 (92.4)	7,043 (92.1)	4,240 (92.2)	5,689 (92.4)	5,760 (92.4)	5,460 (92.9)	0.478
Yes	2,330 (7.6)	607 (7.9)	360 (7.8)	469 (7.6)	476 (7.6)	418 (7.1)	
**Diabetes, %**							
No	28,932 (94.8)	7,233 (94.5)	4,369 (95.0)	5,851 (95.0)	5,954 (95.5)	5,525 (94.0)	**0.004**
Yes	1,590 (5.2)	417 (5.5)	231 (5.0)	307 (5.0)	282 (4.5)	353 (6.0)	
**History of vasectomy, %**							
No	20,843 (68.3)	5,178 (67.7)	3,096 (67.3)	4,167 (67.7)	4,280 (68.6)	4,122 (70.1)	**0.007**
Yes	9,679 (31.7)	2,472 (32.3)	1,504 (32.7)	1,991 (32.3)	1,956 (31.4)	1,756 (29.9)	
Lycopene intake, mg/d	7,391.2 ± 7,372.3	5,579.3 ± 6,135.3	6,605.6 ± 6,479.6	7,328.8 ± 7,167.0	8,108.6 ± 7,900.7	9,668.2 ± 8,359.7	**< 0.001**
Calcium intake, mg/d	829.0 ± 457.8	703.8 ± 432.0	791.8 ± 439.0	827.7 ± 446.1	873.4 ± 438.1	975.2 ± 487.6	**< 0.001**
Vitamin E intake, mg/d	9.9 ± 5.8	7.4 ± 4.7	9.0 ± 5.3	9.7 ± 5.3	10.8 ± 5.6	13.1 ± 6.5	**< 0.001**

DHQ, Dietary Questionnaire; PLCO, Prostate, Lung, Colorectal, and Ovarian Cancer Screening Trial.

The variables are presented as the means ± SDs for continuous variables or percentages for categorical variables. Calcium and vitamin E intakes were calculated from the DHQ. Continuous variables were analyzed by ANOVA.

Univariate analysis of the association between Omega-3 fatty acid intake and prostate cancer risk. Table 2 illustrates the average daily intake (g/d) of Omega-3 fatty acid (EPA, DPA, DHA) among the prostate cancer patients, which included the non-advanced and advanced prostate cancer patients, as well as the non-prostate cancer patients. Significant differences in Omega-3 (EPA, DPA, and DHA) intake were found between the prostate cancer group and the non-prostate cancer group, as well as between advanced prostate cancer patients and the non-prostate cancer group (*P* < 0.05). Conversely, no statistically significant difference in Omega-3 (EPA, DPA, or DHA) intake was observed between the non-advanced prostate cancer group and the non-prostate cancer group (*P* > 0.05).

**Table 2:** Differences in dietary intakes of total Omega-3 (EPA, DPA, DHA) between subjects who did or did not develop prostate cancer in the PLCO screening trial.

**Variable, g/d**	**Total prostate cancer**	**Non-advanced prostate cancer**	**Advanced prostate cancer**	**Non-prostate cancer**	***P***		
					**Total prostate cancer vs. non-prostate cancer**	**Non-advanced prostate cancer vs. non-prostate cancer**	**Advanced prostate cancer vs. non-prostate cancer**
*n*	3,357	2,969	338	27,165			
Omega-3	0.07 (0.04, 0.13)	0.07 (0.04, 0.13)	0.07 (0.04, 0.12)	0.07 (0.04, 0.13)	0.024	0.111	0.019
EPA	0.02 (0.01, 0.04)	0.02 (0.01, 0.04)	0.02 (0.01, 0.03)	0.02 (0.01, 0.04)	0.035	0.133	0.028
DPA	0.01 (0.01, 0.02)	0.01 (0.01, 0.02)	0.01 (0.00, 0.02)	0.01 (0.01, 0.02)	0.048	0.185	0.023
DHA	0.05 (0.03, 0.08)	0.05 (0.03, 0.08)	0.04 (0.03, 0.08)	0.05 (0.03, 0.08)	0.022	0.09	0.027

Omega-3, Omega-3 fatty acids; EPA, eicosapentaenoic acid; DPA, docosapentaenoic acid; DHA, docosahexaenoic acid; DHQ, Dietary History Questionnaire; PLCO, Prostate, Lung, Colorectal, and Ovarian Cancer Screening Trail.

The Wilcoxon rank sum test was used to compare differences in Omega-3 fatty acid intake between subjects with prostate cancer, non-advanced prostate cancer, and advanced prostate cancer during follow-up and those without prostate cancer.

As depicted in Table 3, the multivariate analysis of Omega-3 fatty acid intake and the risk of prostate cancer revealed the relationship between Omega-3 (EPA, DPA, DHA) fatty acid intake and the hazard ratio (HR) of prostate cancer after the intake was categorized into five groups via the five-point method. Without adjusting for covariates, increased intake of EPA and DPA can reduce the risk ratio of prostate cancer. EPA intake in the Q3 group (HR_1_ = 0.90, *P*_1_ = 0.034) and DPA intake in the Q4 group (HR_1_ = 0.86, *P*_1_ = 0.018) were significantly associated with a greater incidence of prostate cancer (HR_1_). However, this effect was no longer observed after adjusting for covariates such as age (HR_2_, HR_3_).

**Table 3:** Hazard ratios (HR) [95% confidence interval (CI)] for prostate cancer by quintiles (Q) of intake of total Omega-3 (EPA, DPA, DHA), assessed using the Dietary History Questionnaire (DHQ), in the intervention arm and control arm of the Prostate, Lung, Colorectal, and Ovarian Cancer Screening Trial.

**Variable (g/d)**	**Cases of prostate cancer**	**Person-years**	**HR_1_**	***P*_1_**	**HR_2_**	***P*_2_**	**HR_3_**	***P*_3_**
**Omega-3**								
Q1 ( ≤ 0.04)	880	90,123		Reference		Reference		Reference
Q2 (>0.04 to ≤ 0.06)	501	54,298	0.94 (0.85–1.05)	0.3	0.96 (0.86–1.07)	0.48	0.97 (0.87–1.08)	0.547
Q3 (>0.06 to ≤ 0.09)	710	72,205	1.01 (0.91–1.11)	0.914	1.04 (0.94–1.15)	0.465	1.05 (0.95–1.16)	0.38
Q4 (>0.09 to ≤ 0.15)	658	73,296	0.92 (0.83–1.02)	0.095	0.96 (0.86–1.06)	0.397	0.97 (0.87–1.07)	0.538
Q5 (>0.15 to ≤ 1.94)	608	68,745	0.90 (0.81–1.00)	0.053	0.97 (0.87–1.07)	0.544	0.99 (0.88–1.10)	0.836
**EPA**								
Q1 ( ≤ 0.01)	1,394	144,304		Reference		Reference		Reference
Q2 (>0.01 to ≤ 0.02)	725	74,684	1.00 (0.92–1.10)	0.928	1.03 (0.94–1.13)	0.489	1.04 (0.95–1.14)	0.423
Q3 (>0.02 to ≤ 0.04)	626	71,662	0.90 (0.82–0.99)	0.034	0.94 (0.85–1.03)	0.187	0.95 (0.86–1.04)	0.252
Q4 (>0.04 to ≤ 0.67)	612	68,017	0.93 (0.85–1.02)	0.133	0.98 (0.89–1.08)	0.723	1.00 (0.90–1.10)	0.946
**DPA**								
Q1 (< 0.01)	831	86,377		Reference		Reference		Reference
Q2 (≥0.01 to < 0.02)	1603	169,849	0.98 (0.90–1.06)	0.623	1.01 (0.93–1.10)	0.763	1.02 (0.94–1.11)	0.631
Q3 (≥0.02 to < 0.03)	573	60,302	0.99 (0.89–1.10)	0.781	1.04 (0.93–1.16)	0.499	1.05 (0.94–1.17)	0.375
Q4 (≥0.03 to ≤ 0.22)	350	42,139	0.86 (0.76–0.97)	0.018	0.94 (0.83–1.07)	0.336	0.96 (0.84–1.10)	0.587
**DHA**								
Q1 ( ≤ 0.03)	1199	124,490		Reference		Reference		Reference
Q2 (>0.03 to ≤ 0.04)	458	45,191	1.05 (0.94–1.17)	0.364	1.07 (0.96–1.19)	0.227	1.08 (0.96–1.20)	0.19
Q3 (>0.04 to ≤ 0.06)	606	66,598	0.94 (0.86–1.04)	0.244	0.97 (0.88–1.07)	0.52	0.98 (0.88–1.08)	0.642
Q4 (>0.06 to ≤ 0.09)	516	57,492	0.93 (0.84–1.03)	0.168	0.97 (0.87–1.07)	0.526	0.98 (0.88–1.09)	0.696
Q5 (>0.09 to ≤ 1.09)	578	64,896	0.92 (0.84–1.02)	0.111	0.98 (0.89–1.09)	0.738	1.00 (0.90–1.12)	0.941

Cox proportional hazards regression analysis was used to calculate hazard ratios (HR) and 95% confidence intervals (CIs) for prostate cancer risk associated with Omega-3 (EPA, DPA, DHA) intake.

EPA and DPA overlapped Q4 and Q5 after categorization according to a five-point scale and were therefore divided into 4 categories.

HR_1_, not adjusted for covariates.

HR_2_, adjusted for age, race, education, marital status, family history of prostate cancer, and randomized trial grouping.

HR_3_, adjusted for age, race, education, marital status, family history of prostate cancer, randomized trial subgroup, BMI, height, history of diabetes mellitus, history of vasectomy, smoking in the past 10 years, energy-adjusted lycopene, calcium, and vitamin E intake.

Table 4 presents the multivariate analysis of Omega-3 fatty acid intake and the risk of prostate cancer death, specifically the relationship between Omega-3 (EPA, DPA, DHA) fatty acid intake, categorized into five groups via the five-point method, and the hazard ratio (HR) of prostate cancer death. Without adjusting for covariates, increasing Omega-3 (EPA, DPA, and DHA) intake can reduce the risk of prostate cancer death, and the greater the intake is, the greater the reduction effect (HR_1_). Even after adjusting for covariates such as age (HR_3_), increasing Omega-3 intake and EPA intake can significantly reduce the risk of prostate cancer death.

**Table 4:** Hazard ratios (HRs) [95% confidence intervals (CIs)] for prostate cancer deaths in the intervention and control groups of the prostate, lung, colorectal, and ovarian cancer screening trial intervention and control groups for quintiles (Q) of total Omega-3 (EPA, DPA, and DHA) intake as assessed by the Dietary History Questionnaire (DHQ).

**Variable (g/d)**	**Numbers of prostate cancer deaths**	**Person-years**	**HR_1_**	***P*_1_**	**HR_2_**	***P*_2_**	**HR_3_**	***P*_3_**
**Omega-3**								
Q1 ( ≤ 0.04)	146	61,541	Reference		Reference		Reference	
Q2 (>0.04 to ≤ 0.06)	54	37,831	0.61 (0.44–0.83)	0.002	0.69 (0.50–0.94)	0.018	0.67 (0.49–0.91)	0.011
Q3 (>0.06 to ≤ 0.09)	93	52,020	0.74 (0.57–0.97)	0.026	0.85 (0.66–1.11)	0.23	0.83 (0.64–1.08)	0.174
Q4 (>0.09 to ≤ 0.15)	87	52,635	0.70 (0.53–0.91)	0.008	0.84 (0.64–1.09)	0.192	0.80 (0.61–1.05)	0.113
Q5 (>0.15 to ≤ 1.94)	72	50,038	0.60 (0.45–0.80)	< 0.001	0.76 (0.57–1.00)	0.054	0.70 (0.52–0.95)	0.021
**EPA**								
Q1 ( ≤ 0.01)	208	99,507	Reference		Reference		Reference	
Q2 (>0.01 to ≤ 0.02)	91	53,677	0.80 (0.63–1.03)	0.078	0.89 (0.69–1.13)	0.334	0.88 (0.68–1.12)	0.301
Q3 (>0.02 to ≤ 0.04)	83	51,128	0.78 (0.60–1.00)	0.054	0.90 (0.69–1.16)	0.397	0.88 (0.68–1.14)	0.316
Q4 (>0.04 to ≤ 0.67)	70	49,753	0.66 (0.50–0.87)	0.003	0.78 (0.59–1.02)	0.073	0.74 (0.56–0.99)	0.041
**DPA**								
Q1 (< 0.01)	136	58,717	Reference		Reference		Reference	
Q2 (≥0.01 to < 0.02)	201	120,881	0.71 (0.57–0.89)	0.002	0.85 (0.68–1.05)	0.133	0.83 (0.67–1.04)	0.101
Q3 (≥0.02 to < 0.03)	76	44,068	0.73 (0.55–0.97)	0.029	0.92 (0.69–1.22)	0.551	0.88 (0.66–1.18)	0.408
Q4 (≥0.03 to ≤ 0.22)	39	30,399	0.56 (0.39–0.79)	0.001	0.77 (0.54–1.11)	0.16	0.71 (0.49–1.04)	0.078
**DHA**								
Q1 ( ≤ 0.03)	177	85,533	Reference		Reference		Reference	
Q2 (>0.03 to ≤ 0.04)	60	32,398	0.88 (0.65–1.18)	0.379	0.93 (0.69–1.25)	0.635	0.91 (0.68–1.23)	0.544
Q3 (>0.04 to ≤ 0.06)	76	47,395	0.77 (0.59–1.01)	0.063	0.87 (0.66–1.14)	0.303	0.85 (0.65–1.12)	0.254
Q4 (>0.06 to ≤ 0.09)	67	41,539	0.79 (0.59–1.04)	0.093	0.91 (0.69–1.21)	0.536	0.88 (0.66–1.17)	0.382
Q5 (>0.09 to ≤ 1.09)	72	47,200	0.73 (0.55–0.96)	0.024	0.89 (0.67–1.17)	0.399	0.83 (0.62–1.12)	0.222

Hazard ratios (HR) and 95% confidence intervals (CI) for the risk of prostate cancer death associated with Omega-3 (EPA, DPA, DHA) intake were calculated using Cox proportional hazards regression analysis.

EPA and DPA overlapped Q4 and Q5 after categorization according to a five-point scale and were therefore divided into 4 categories.

HR_1_, not adjusted for covariates.

HR_2_, adjusted for age, race, education, marital status, family history of prostate cancer, and randomized trial grouping.

HR_3_, adjusted for age, race, education, marital status, family history of prostate cancer, randomized trial subgroup, BMI, height, history of diabetes mellitus, history of vasectomy, smoking in the past 10 years, energy-adjusted lycopene, calcium, and vitamin E intake.

**Figure 2:**
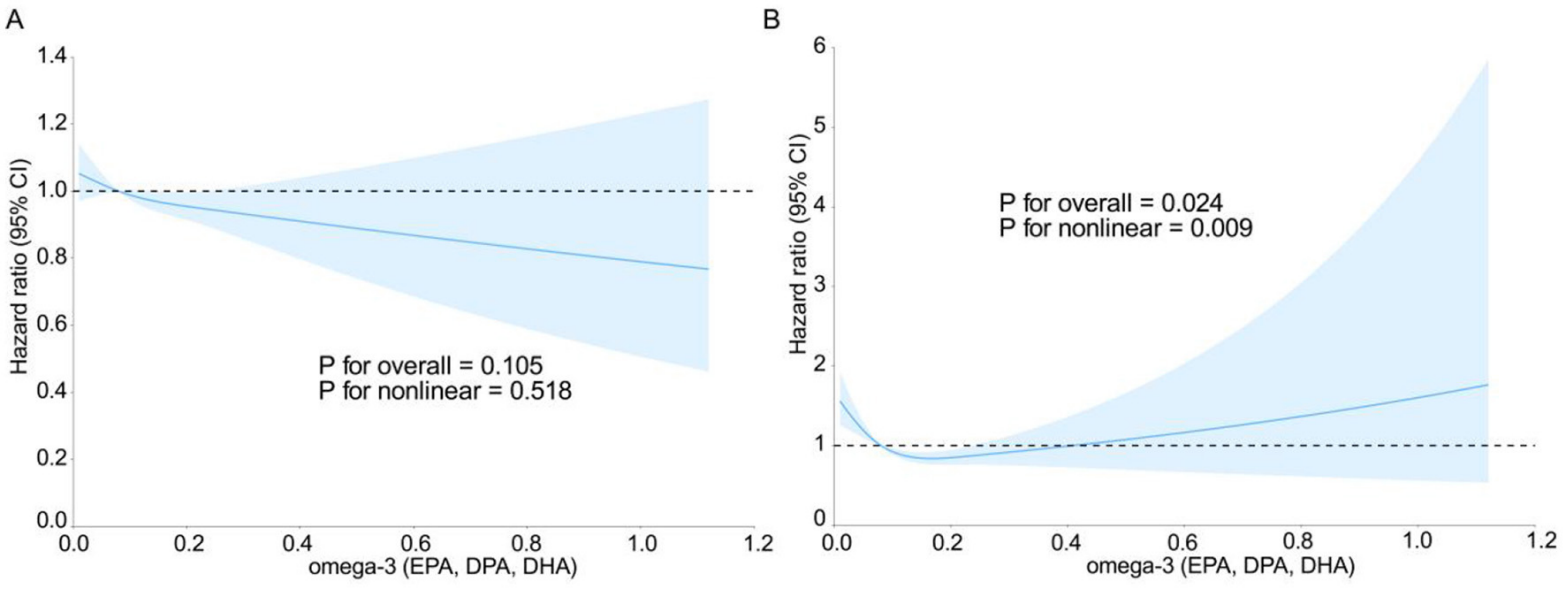
Restricted cubic spline curves of prostate cancer risk ratios **(A)** and prostate cancer mortality risk ratios **(B)** vs. total Omega-3 (EPA, DPA, DHA) intake in the intervention and control groups of the prostate, lung, colorectal, and ovarian cancer screening trial.

Figure 2 shows the relationships between Omega-3 fatty acid intake and the risk of prostate cancer and death. After adjusting for covariates, including age, race, education, marital status, family history of prostate cancer, randomized trial subgroup, BMI, height, history of diabetes mellitus, history of vasectomy, smoking in the past 10 years, and energy-adjusted lycopene, calcium, and vitamin E intake, a linear relationship was observed between Omega-3 intake and the hazard ratio (HR) of prostate cancer (*P*_non-*linearity*_ = 0.518) (Figure 2A). As the intake of Omega-3 fatty acids increases, the risk of prostate cancer gradually decreases. There was a non-linear relationship between Omega-3 fatty acid intake and the hazard ratio (HR) of prostate cancer death (*P*_non-*linearity*_ = 0.009) (Figure 2B). The RCS analysis revealed a potential U-shaped association for mortality risk, with the lowest risk corresponding to an intake range of ~0.15–0.40 g/d. When the intake of Omega-3 fatty acids is < 0.4 g/d, it is beneficial for reducing the risk of prostate cancer death, whereas when the intake of Omega-3 fatty acids is >0.4 g/d, it increases the risk of prostate cancer death.

## Discussion

By utilizing extensive population data from the PLCO trial, this study investigated the relationships between Omega-3 fatty acid intake and the risk of prostate cancer and death. The results revealed that Omega-3 fatty acid intake was linearly related to the overall risk of prostate cancer, whereas a non-linear relationship was observed with the risk of prostate cancer death.

It is evident that the role of Omega-3 fatty acids in prostate cancer is significant and cannot be overlooked. Our findings agree with the results of Claire Bosire's index-based dietary patterns and prostate cancer risk in the AARP Diet and Health Study (20) and Katarina Augustsson's prospective study on fish and marine fatty acid intake and prostate cancer (21); that is, higher intake of Omega-3 can reduce the risk of prostate cancer. Our study specifically focused on elucidating the relationship between Omega-3 fatty acid intake and the risk of both prostate cancer and death. Our results revealed that there may be a certain association between Omega-3 (EPA, DPA, DHA) intake and the risk of prostate cancer. In the multivariate analysis of Omega-3 fatty acid intake and prostate cancer, the original effect, that is, the greater the intake, the lower the risk of prostate cancer, disappeared after adjusting for a series of covariates. However, in the RCS analysis, we found that the greater the level of Omega-3 fatty acid intake was, the lower the risk of prostate cancer. In terms of the relationship with the risk of death, we found that when the intake of Omega-3 fatty acids is < 0.4 g/d, it is beneficial for reducing the risk of death from prostate cancer, and when the intake of Omega-3 fatty acids is >0.4 g/d, it increases the risk of death from prostate cancer. These findings suggest that in providing dietary treatment for prostate cancer, the progression of prostate cancer can be alleviated by adjusting the intake of Omega-3 fatty acids. We speculate that when the intake of Omega-3 fatty acids exceeds 0.4 g/d, the harmful effect on the body may be because it has a certain impact on the hormone levels in the body, causing hormone imbalance and thus leading to cell cycle disorders, increasing the risk of cell cancer. Because in Manni et al.'s (22) study on the combination of anti-estrogen and Omega-3 fatty acids to prevent breast cancer, it was found from rat experiments that Omega-3 can affect the changes in estrogen and progesterone in the body.

Our research method has produced two seemingly contradictory views on the relationship between Omega-3 fatty acid intake and the risk of prostate cancer. This may be because the proportion of patients without prostate cancer in the included study population is relatively large; in terms of research methods, the types of variables involved in multivariate analysis and RCS analysis are different. Multivariate analysis divides Omega-3 fatty acid intake into quintiles, which is a categorical variable, which may lead to the loss of continuous information of intake and make it difficult to capture subtle dose-response relationships. In addition, the sample size of the high-intake group is small, the statistical power is reduced, and it is difficult to detect weak but consistent effects. RCS analysis regards Omega-3 intake as a continuous variable, and flexibly models it through cubic spline functions, which retains the continuity of intake, can more sensitively detect overall trends, and test linear and non-linear relationships at the same time. These two results are actually complementary. Multivariate analysis shows no significant difference between groups after adjustment, which may be due to information loss caused by grouping; The RCS analysis reveals the overall linear trend through continuous modeling, indicating that there is a weak but consistent negative correlation between intake and risk, but owing to the small effect size, it failed to show up in the group analysis, thus filling this limitation in the multivariate analysis.

In contrast to the risk of prostate cancer, the multivariate analysis of Omega-3 intake and the risk of prostate cancer death demonstrated that increasing Omega-3 (EPA, DPA, DHA) intake could reduce the risk of prostate cancer death, irrespective of whether relevant variables such as age were adjusted for. As a polyunsaturated fatty acid (PUFA), Omega-3 fatty acids influence prostate cancer primarily through the COX enzyme (23). In a study by Fradet et al. (24) on Omega-3 fatty acids and COX-2 genetic variation, COX-2 was found to be excessive in prostate cancer. As the core enzyme of eicosanoid synthesis, COX-2 overproduction leads to an increase in the levels of proinflammatory eicosanoids in the body, which leads to increased inflammation. However, eicosanoids derived from Omega-3 fatty acids have anti-inflammatory activity and have an inhibitory effect on the overproduction of COX-2, thereby alleviating the risk and progression of prostate cancer ^3^. Omega-3 can also reduce the synthesis of proinflammatory cytokines (such as IL-6 and TNF-α) and prostaglandin E2 (PGE2), which also works through the COX pathway and competitively antagonizes Omega-6 fatty acids to reduce the production of proinflammatory eicosanoids, thereby improving immune surveillance in the cancer microenvironment. In addition, omega-3 fatty acids induce cancer cell apoptosis by accumulating reactive oxygen species (ROS) and activating caspases, and also activate autophagy flux through SIRT1-mediated Beclin-1 deacetylation and AMPK pathway, thereby alleviating oxidative stress and inflammation (25, 26). These mechanisms work together to make Omega-3 fatty acids have potential application value in cancer treatment and tissue protection.

Moreover, in the multivariate analysis of the risk of prostate cancer, prior to adjusting for variables, EPA intake in the Q3 group (HR_1_ = 0.90, *P*_1_ = 0.034) and DPA intake in the Q4 group (HR_1_ = 0.86, P_1_ = 0.018) exhibited a statistically significant trend toward reducing prostate cancer risk. This finding indicates that increasing the intake of specific Omega-3 components (EPAs, DPAs) through dietary adjustments could serve as a strategy for prostate cancer prevention, particularly for high-risk groups such as those with a family history or obesity. Targeted supplementation may hold greater value in such cases. Therefore, we recommend that healthy people consume foods rich in Omega-3 fatty acids, such as deep-sea fish, fish oil, and certain seaweeds and fermented foods (such as natto). Combined with the results of Omega-3 intake and the risk of death from prostate cancer, we recommend that the daily intake of healthy people should be within a certain range, such as ~0.02–0.04 g/d for EPA and ~0.03–0.22 g/d for DPA, which can be achieved by consuming deep-sea fish at least twice a week (~100–150 g each time) or taking appropriate supplements. In addition, threshold awareness, that is, with respect to the results of Omega-3 and the risk of death from prostate cancer, the total Omega-3 intake needs to be controlled below 0.4 g/d to balance the risks and benefits. There is still no recognized clinical guideline for this specific intake recommendation, and there are relatively few studies supporting this intake, so the recommendations of this study are only made on the basis of this result.

Notably, the strengths of this study lie in its large-scale population data and long-term follow-up, which provide robust epidemiological evidence. Additionally, this study elucidated the dietary relationship between Omega-3 fatty acids and prostate cancer via the PLCO and DHQ databases, thereby filling the gap in the lack of reliable dietary intake recommendations for the dietary treatment of prostate cancer. Moreover, the introduction of a ketogenic diet as a weight loss therapy has expanded the application scenarios of dietary interventions in prostate cancer management. Moreover, we adjusted for a variety of potential confounders, including age, race, BMI, smoking history, family history, and dietary nutrients (lycopene, calcium, vitamin E), to reduce the impact of residual confounding. This study innovatively combined the study of categorical variables and continuous variables, complemented the results, made our research results more reliable, and introduced RCS analysis to reveal the complex dose–response pattern of Omega-3 intake and prostate cancer risk. However, the study also has several limitations. First, the collection of dietary data was limited to the baseline DHQ, which did not account for potential dietary changes during the follow-up period. Second, as the DHQ is self-reported, it is subject to certain subjective factors, which may compromise the accuracy of the information and introduce recall bias. In addition, the subjects of this study were mainly non-Hispanic White men in the United States aged 55–74 years, and the results may not be applicable to young men, other races or regions. In addition, because this study explored only the intake of Omega-3 fatty acids as the sum of the contents of three substances, DHA, DPA and EPA, the effect of Omega-3 fatty acids may be affected by other nutrients (such as Omega-6, vitamin D, and vitamin E) (27–29). Although we adjusted for multiple confounding factors, residual confounding may still affect the results. Moreover, group analysis may lead to the loss of dose–response information, especially for the high-dose group (such as Q5, which covers 0.15–1.94 g/d), which has high heterogeneity, diluting the potential effect.

In future studies, multiple dietary assessments (such as the regular FFQ or biomarker testing) can be conducted in cohort studies to analyze the relationship between changes in Omega-3 fatty acid intake and prostate cancer progression dynamically, especially the cumulative effect of long-term intake on prostate cancer survival. In addition, randomized controlled trials (RCTs) can be designed to evaluate the direct effects of Omega-3 supplements or ketogenic diet interventions on the prognosis of prostate cancer patients, with a focus on dose effects, safety, and compliance. In summary, future studies can further explore the impact of Omega-3 fatty acids on the dietary level of patients with prostate cancer.

## Conclusion

On the basis of the data from the PLCO trial, this study found that Omega-3 fatty acid intake is linearly negatively correlated with the overall risk of prostate cancer, that is, the higher the intake, the lower the risk. However, it is non-linearly related to the risk of death from prostate cancer, and there is a threshold effect. When the intake exceeds 0.4 g/d, it may increase the risk of death. The data suggest a potential threshold effect around 0.4 g/d, above which Omega-3 intake may be associated with increased prostate cancer mortality. However, further investigation is needed to confirm this threshold and its clinical relevance.

## Data Availability

The raw data supporting the conclusions of this article will be made available by the authors, without undue reservation.

## References

[1] Bergengren O Pekala KR Matsoukas K Fainberg J Mungovan SF Bratt O. 2022 update on prostate cancer epidemiology and risk factors-a systematic review. Eur Urol. (2023) 84:191–206. 10.1016/j.eururo.2023.04.021 37202314 PMC10851915

[2] Bray F Laversanne M Sung H Ferlay J Siegel RL Soerjomataram I. Global cancer statistics 2022: GLOBOCAN estimates of incidence and mortality worldwide for 36 cancers in 185 countries. CA Cancer J Clin. (2024) 74:229–63. 10.3322/caac.21834 38572751

[3] Leslie SW Soon-Sutton TL Skelton WP. Prostate Cancer. In: StatPearls [Internet]. Treasure Island (FL): StatPearls Publishing (2025). 29261872

[4] Ubago-Guisado E Rodríguez-Barranco M Ching-López A Petrova D Molina-Montes E Amiano P. Evidence update on the relationship between diet and the most common cancers from the European Prospective Investigation into Cancer and Nutrition (EPIC) Study: a systematic review. Nutrients. (2021) 13:3582. 10.3390/nu13103582 34684583 PMC8540388

[5] Wang Y Liu K Long T Long J Li Y Li J. Dietary fish and omega-3 polyunsaturated fatty acids intake and cancer survival: a systematic review and meta-analysis. Crit Rev Food Sci Nutr. (2023) 63:6235–51. 10.1080/10408398.2022.2029826 35068276

[6] Shahidi F Ambigaipalan P. Omega-3 polyunsaturated fatty acids and their health benefits. Annu Rev Food Sci Technol. (2018) 9:345–81. 10.1146/annurev-food-111317-095850 29350557

[7] Calder PC. n-3 PUFA and inflammation: from membrane to nucleus and from bench to bedside. Proc Nutr Soc. (2020) 22:1–13. 10.1017/S0029665120007077 32624016

[8] Deckelbaum RJ Worgall TS Seo T. n-3 fatty acids and gene expression. Am J Clin Nutr. (2006) 83:1520S−5S. Erratum in: *Am J Clin Nutr*. (2006) 84:949. 10.1093/ajcn/83.6.1520S 16841862

[9] D'Angelo S Motti ML Meccariello R. ω-3 and ω-6 polyunsaturated fatty acids, obesity and cancer. Nutrients. (2020) 12:2751. 10.3390/nu12092751 32927614 PMC7551151

[10] Kim H Kim JK. Evidence on statins, omega-3, and prostate cancer: a narrative review. World J Mens Health. (2022) 40:412–24. 10.5534/wjmh.210139 35021299 PMC9253794

[11] Kousparou C Fyrilla M Stephanou A Patrikios I. DHA/EPA (Omega-3) and LA/GLA (Omega-6) as bioactive molecules in neurodegenerative diseases. Int J Mol Sci. (2023) 24:10717. 10.3390/ijms241310717 37445890 PMC10341783

[12] Qian F Tintle N Jensen PN Lemaitre RN Imamura F Feldreich TR. Omega-3 fatty acid biomarkers and incident atrial fibrillation. J Am Coll Cardiol. (2023) 82:336–49. 10.1016/j.jacc.2023.05.024 37468189

[13] Weylandt KH Serini S Chen YQ Su HM Lim K Cittadini A. Omega-3 polyunsaturated fatty acids: the way forward in times of mixed evidence. Biomed Res Int. (2015) 2015:143109. 10.1155/2015/143109 26301240 PMC4537707

[14] Brasky TM Till C White E Neuhouser ML Song X Goodman P. Serum phospholipid fatty acids and prostate cancer risk: results from the prostate cancer prevention trial. Am J Epidemiol. (2011) 173:1429–39. 10.1093/aje/kwr027 21518693 PMC3145396

[15] Brasky TM Darke AK Song X Tangen CM Goodman PJ Thompson IM. Plasma phospholipid fatty acids and prostate cancer risk in the SELECT trial. J Natl Cancer Inst. (2013) 105:1132–41. 10.1093/jnci/djt174 23843441 PMC3735464

[16] Szymanski KM Wheeler DC Mucci LA. Fish consumption and prostate cancer risk: a review and meta-analysis. Am J Clin Nutr. (2010) 92:1223–33. 10.3945/ajcn.2010.29530 20844069

[17] Alexander DD Bassett JK Weed DL Barrett EC Watson H Harris W. Meta-analysis of Long-Chain Omega-3 Polyunsaturated Fatty Acids (LCω-3PUFA) and prostate cancer. Nutr Cancer. (2015) 67:543–54. 10.1080/01635581.2015.1015745 25826711 PMC4440629

[18] Shikany JM Flood AP Kitahara CM Hsing AW Meyer TE Willcox BJ. Dietary carbohydrate, glycemic index, glycemic load, and risk of prostate cancer in the Prostate, Lung, Colorectal, and Ovarian Cancer Screening Trial (PLCO) cohort. Cancer Causes Control. (2011) 22:995–1002. 10.1007/s10552-011-9772-1 21553078 PMC4470253

[19] Hoyt M Reger M Marley A Fan H Liu Z Zhang J. Vitamin K intake and prostate cancer risk in the Prostate, Lung, Colorectal, and Ovarian Cancer (PLCO) screening trial. Am J Clin Nutr. (2019) 109:392–401. 10.1093/ajcn/nqy251 30624568

[20] Bosire C Stampfer MJ Subar AF Park Y Kirkpatrick SI Chiuve SE. Index-based dietary patterns and the risk of prostate cancer in the NIH-AARP diet and health study. Am J Epidemiol. (2013) 177:504–13. 10.1093/aje/kws261 23408548 PMC3657529

[21] Augustsson K Michaud DS Rimm EB Leitzmann MF Stampfer MJ Willett WC. A prospective study of intake of fish and marine fatty acids and prostate cancer. Cancer Epidemiol Biomarkers Prev. (2003) 12:64–7. 12540506

[22] Manni A El-Bayoumy K Skibinski CG Thompson HJ Santucci-Pereira J Bidinotto LT. Combination of antiestrogens and Omega-3 fatty acids for breast cancer prevention. Biomed Res Int. (2015) 2015:638645. 10.1155/2015/638645 26339626 PMC4538406

[23] Reese AC Fradet V Witte JS. Omega-3 fatty acids, genetic variants in COX-2 and prostate cancer. J Nutrigenet Nutrigenom. (2009) 2:149–58. 10.1159/000235565 19776642 PMC2820568

[24] Fradet V Cheng I Casey G Witte JS. Dietary Omega-3 fatty acids, cyclooxygenase-2 genetic variation, and aggressive prostate cancer risk. Clin Cancer Res. (2009) 15:2559–66. 10.1158/1078-0432.CCR-08-2503 19318492 PMC2749066

[25] Chen X Pan Z Fang Z Lin W Wu S Yang F. Omega-3 polyunsaturated fatty acid attenuates traumatic brain injury-induced neuronal apoptosis by inducing autophagy through the upregulation of SIRT1-mediated deacetylation of Beclin−1. J Neuroinflamm. (2018) 15:310. 10.1186/s12974-018-1345-8 30409173 PMC6225685

[26] Montecillo-Aguado M Tirado-Rodriguez B Huerta-Yepez S. The involvement of polyunsaturated fatty acids in apoptosis mechanisms and their implications in cancer. Int J Mol Sci. (2023) 24:11691. 10.3390/ijms241411691 37511450 PMC10380946

[27] Zhang Y Sun Y Yu Q Song S Brenna JT Shen Y. Higher ratio of plasma omega-6/omega-3 fatty acids is associated with greater risk of all-cause, cancer, and cardiovascular mortality: a population-based cohort study in UK Biobank. Elife. (2024) 12:RP90132. 10.7554/eLife.90132 38578269 PMC10997328

[28] Bischoff-Ferrari HA Gängler S Wieczorek M Belsky DW Ryan J Kressig RW. Individual and additive effects of vitamin D, omega-3 and exercise on DNA methylation clocks of biological aging in older adults from the DO-HEALTH trial. Nat Aging. (2025) 5:376–85. 10.1038/s43587-024-00793-y 39900648 PMC11922767

[29] Sadeghi F Alavi-Naeini A Mardanian F Ghazvini MR Mahaki B. Omega-3 and vitamin E co-supplementation can improve antioxidant markers in obese/overweight women with polycystic ovary syndrome. Int J Vitam Nutr Res. (2020) 90:477–83. 10.1024/0300-9831/a000588 30961460

